# Removal Dynamics of Nitric Oxide (NO) Pollutant Gas by Pulse-Discharged Plasma Technique

**DOI:** 10.1155/2014/653576

**Published:** 2014-03-05

**Authors:** Lianshui Zhang, Xiaojun Wang, Weidong Lai, Xueliang Cheng, Kuifang Zhao

**Affiliations:** College of Physics Science and Technology, Hebei University, Baoding, Hebei Province 071002, China

## Abstract

Nonthermal plasma technique has drawn extensive attentions for removal of air pollutants such as NO_*x*_ and SO_2_. The NO removal mechanism in pulse discharged plasma is discussed in this paper. Emission spectra diagnosis indicates that the higher the discharge voltage is, the more the NO are removed and transformed into O, N, N_2_, NO_2_, and so forth. Plasma electron temperature *T*_*e*_ is ranged from 6400 K at 2.4 kV discharge voltage to 9500 K at 4.8 kV. After establishing a zero-dimensional chemical reaction kinetic model, the major reaction paths are clarified as the electron collision dissociation of NO into N and O during discharge and followed by single substitution of N on NO to form N_2_ during and after discharge, compared with the small fraction of NO_2_ formed by oxidizing NO. The reaction directions can be adjusted by N_2_ additive, and the optimal N_2_/NO mixing ratio is 2 : 1. Such a ratio not only compensates the disadvantage of electron competitive consumption by the mixed N_2_, but also heightens the total NO removal extent through accelerating the NO oxidization process.

## 1. Introduction

Nitrogen Oxides (NO_*x*_), usually emitted through exhaust gas, have assumed the major responsibility with sulfur dioxide (SO_2_) for acid rain formation, which is harmful to human health and to the environment through acid deposition [1–3]. High-efficient NO_*x*_ remediation or removal technologies has drawn extensive attentions for environmental protection from the viewpoint of fuel corporations, thermal power plants, explosive motor designers, and so on [4–7]. Catalysis reduction [8–11] or direct thermal decomposition [12, 13] approaches have been applied, but the reaction temperatures are usually high [14], which cannot directly be used for removing the 100–200°C diesel or power plant exhaust gas. One candidate way without high temperature control is the nonthermal plasma method at atmospheric pressure [15–17]. Such technique is typically consisting of an array of discharge filaments lasting for tens to hundreds of nanoseconds, in which high energy electrons are initiated by the high voltage and flowed into the exhaust gas. Electron collision can decompose the NO_*x*_ into other chemical species as atoms, ions, and molecules, restricted by the law of conservation of matter and the overall charge roughly of zero. Complicated physical-chemical reactions have occurred between the electrons, by-products, and the pollutant NO_*x*_ [18, 19].

Various kinds of NO removal discharge techniques have been developed to date. One key factor that affects the removal efficiency is the appropriate gas components and discharge parameters. Leipold et al. plasma-treated exhaust gas by atmospheric pressure DBD (dielectric barrier discharges) and find that the N atoms have efficiently participated in the reaction of deoxidizing NO to N_2_ and O [27]. Wang et al. regard the hydroxyl (OH), decomposed from H_2_O, as the active radical to increase the oxidation efficiency of NO in NO/O_2_/N_2_/C_2_H_4_ system. When too more vapor is mixed, the quenching effect on NO removal appears through the consumption on other active species by H_2_O [28]. Tsai et al. find that the elevated discharge power or decreased O_2_ concentration can improve the NO removal efficiency for the NO/N_2_/O_2_ (2%)/H_2_O (10%) mixtures [29]. To monitor the de-NO_*x*_ process, optical emission spectroscopy [30, 31] or laser induced fluorescence (LIF) [32] has been applied for contactless diagnosis of the radicals and by-products in plasma.

Another factor is the design of plasma reactor structure for improving energy consumption efficiency. Plasma generated by radio-frequency (RF) electric field is reported to resonantly accumulate electromagnetic energy at low-voltage power supply, with NO efficiently oxidized to form NO_2_ in the plasma at atmospheric pressure [33]. Multirod DBD reactor is introduced for NO removal based on both dielectric barrier discharges and sliding discharges, and higher NO removal rate is yielded by pulse discharge in comparison with AC discharge at same energy density [34]. The influence of surface plasma reactions on NO_*x*_ removal has been recognized; surface- and volume-plasma are compared [35]. In order to remove NO from exhaust gas, investigations are also focused on the conversion of NO into NO_2_, which can be conveniently removed by chemical scrubbers such as sodium sulfite (Na_2_SO_3_) solution [36].

In order to further promote the NO_*x*_ removal, plasma technique can be combined with catalysts. Combined adsorption-discharge plasma technique has been proposed, as NO_*x*_ firstly adsorbed on H-ZSM-5c substrate and then reduced by CH_4_ or NH_3_ in plasma [37, 38]. Takahara et al. report that Pt catalyst can assist the discharged NO removal process with high activity under a humidified condition, in which N_2_O formed as intermediate species for promoting N_2_ formation [39]. Pulsed corona combined with CaOH_2_ absorption technique presents that SO_2_ and NO in the gas are oxidized into SO_3_ and NO_2_ by plasma and then absorbed by the CaOH_2_ [40]. Such combined processes have the advantages of operating in a wide range of temperatures.

Most of the researches pay attentions to the topic about how to achieve higher NO_*x*_ removal efficiency with lower energy cost for practical demand. But the practicability has been restricted by the poor comprehensions of dynamic mechanism of NO removal due to the complicated reactions in plasma. Accurate measurements of the nitrogen oxides and other by-products under different conditions are usually difficult. It is necessary to clarify the NO removal process in order to optimize the operating conditions such as the applied voltage and the additive gas.

In this paper, the pulse discharged plasma for NO remediation has been experimentally achieved and theoretically simulated. Emission spectra are diagnosed to discriminate the category of by-products and supervise the NO removal process. The relationship between discharge voltage and plasma electron temperatures *T*_e_ is discussed to evaluate the chemical activity of NO removal reactions from macroscopic viewpoint. Since the microcosmic dynamic process is difficult to be experimentally obtained, a chemical kinetic model is developed in this paper. Generating and losing reactions of different main species in the plasma have been analyzed. The effects of pulse discharge duration width and additive gas on NO removal efficiency are focused on. It finds that pulse-discharged plasma technique assisted with N_2_ additive can efficiently remove the gas pollutant of NO and has the potential utilization in power plants, automobiles, and so on.

## 2. Experimental Methods

**Figure 1:**
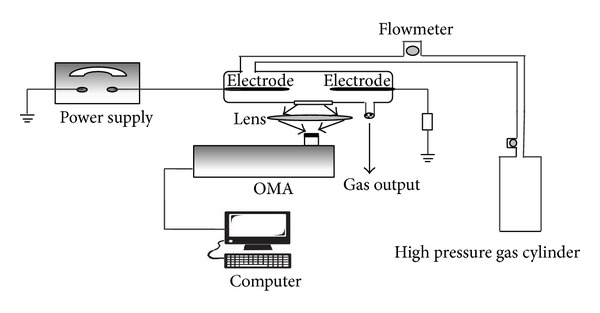
Schematic diagram of the pulse-discharged system for NO removal.

The experimental instrument is schematically present in Figure 1. Two tungsten pointing electrodes of 1 mm in diameter are oppositely placed to act as discharge system, with the separation distance of 3 mm. The two electrodes are powered by high voltage pulse source, which can be adjusted in the range from 1 to 15 kV. The pulse duration is 24.5 ns, with discharge frequency of 50 Hz. Discharge process between the electrodes is operated in a glass reactor with diameter of 25 mm and volumetric capacity of 40 mL. Gases with purity of 99.99% are delivered into the discharge zone after flowing through the connected pipes. The air pressure in the reactor is manipulated by vacuum pump and gas flowmeter and controlled at 0.2 atm.

Emission spectra are collected through a quartz window, with diameter of 18 mm on the glass reactor wall corresponding to the discharge zone. The spectra are then detected by OMA (Optical Multichannel Analyzer, ACTON, SP-2300) with the spectra detecting resolution of 0.1 nm.

## 3. Results and Discussion

There are two critical factors in practical application for discharge removing pollutant gas: (1) electrical energy consumption and (2) by-product identification. Here, the former is evaluated as plasma electron temperature *T*_e_ based on *Boltzmann* distribution theory; the latter is obtained based on the emission spectra diagnostic technique.

### 3.1. Emission Spectra Diagnostics of NO

**Figure 2:**
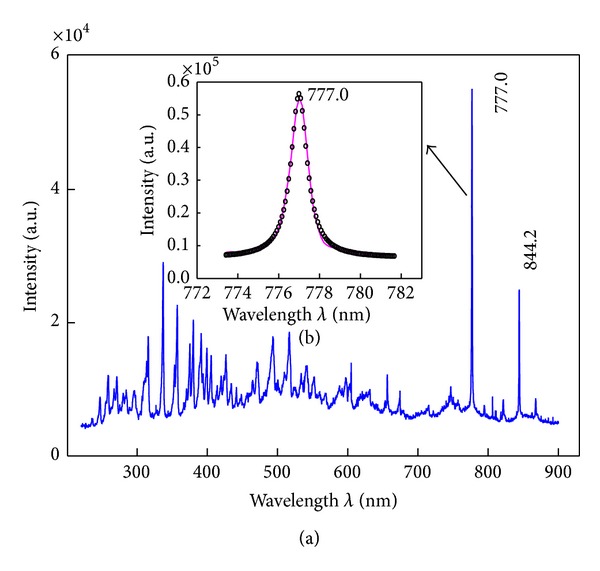
(a) Emission spectra detected from the pulse discharged instrument; (b) *Lorentz* fitting for the emission spectra of O atoms at 777.0 nm.

The NO emission spectra have been detected and shown in Figure 2(a). A notable sequence of UV radiation has been produced, which belongs to NO and new species of N_2_ molecules as well as N_2_^+^ ion. From 493 nm, spectra of another new species NO_2_ appear. In the wavelength range from 656 nm to 867 nm, the atom or ion species of N, O, N^+^ have played the essential roles. The atom spectra of N and O have only instrument broadening and can be well fitted by *Lorentz* function, which has been shown in Figure 2(b) with O atom at 777.0 nm as an example.

Apart from those lines of the NO, the most important feature of the emission diagnostics is that new species of N, N_2_, N^+^, N_2_^+^, and O as well as NO_2_ appeared. O_2_ molecules are also formed, though no distinct emission lines detected, due to its very low transition probability for radiation. It implies that some extent of NO has been transformed into new species, including harmless species such as N, O, N_2_, O_2_, N^+^, and N_2_^+^, or can be further removed by other methods such as NO_2_.

The emission spectra and their transition channels are evaluated [41] and outlined in Table 1.

**Figure 3:**
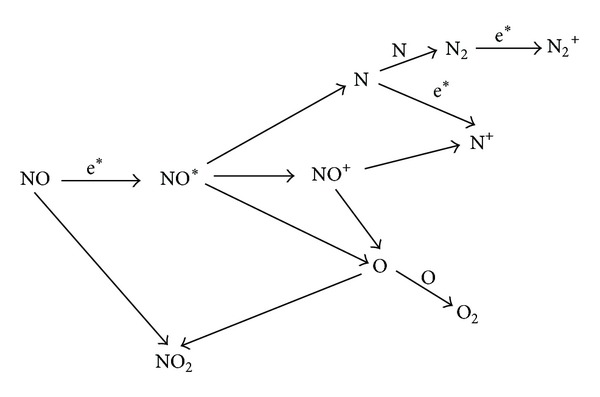
Diagram of main reaction paths and species during discharge.

Based on the species diagnostic details in Table 1, the main reaction paths and chemical species with comparatively large reaction magnitude order are estimated as follows (Figure 3). During discharge between the two electrodes, elastic collision and inelastic collision between the injected electrons and NO molecules have initiated the electron-impact dissociation of NO, followed by other subsequent reactions to form NO^+^, N, N_2_, N_2_^+^, N^+^, O, and O_2_ as well as NO_2_.

Though NO_2_ is generated as another kind of nitrogen oxides which is also harmful, the NO_2_ can be conveniently reduced using sulfite solution based on the reaction as 2NO_2_ + 4SO_3_^−2^ → 4SO_4_^−2^ + N_2_ [42]. NO_2_ generation is usually accepted to be effective for NO removal.

**Figure 4:**
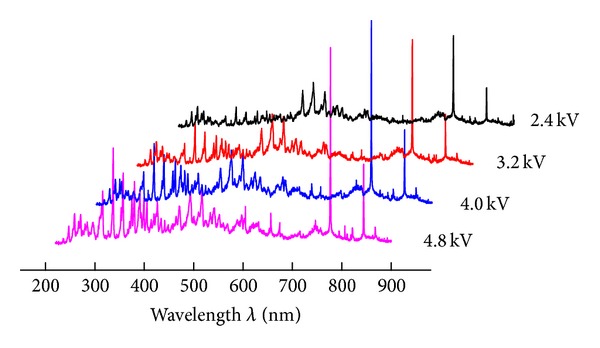
Emission spectra under different pulse voltage inputted from 2.4 to 4.8 kV.

The electrical energy consumption for achieving desirable NO removal efficiency is of critical importance in practice. Under different discharge voltage from 2.4 to 4.8 kV, the emission spectra are shown in Figure 4.

By high voltage loaded, the emission spectra lines are located at the almost unchanged wavelength. Differently, the emission intensity has varied.

**Figure 5:**
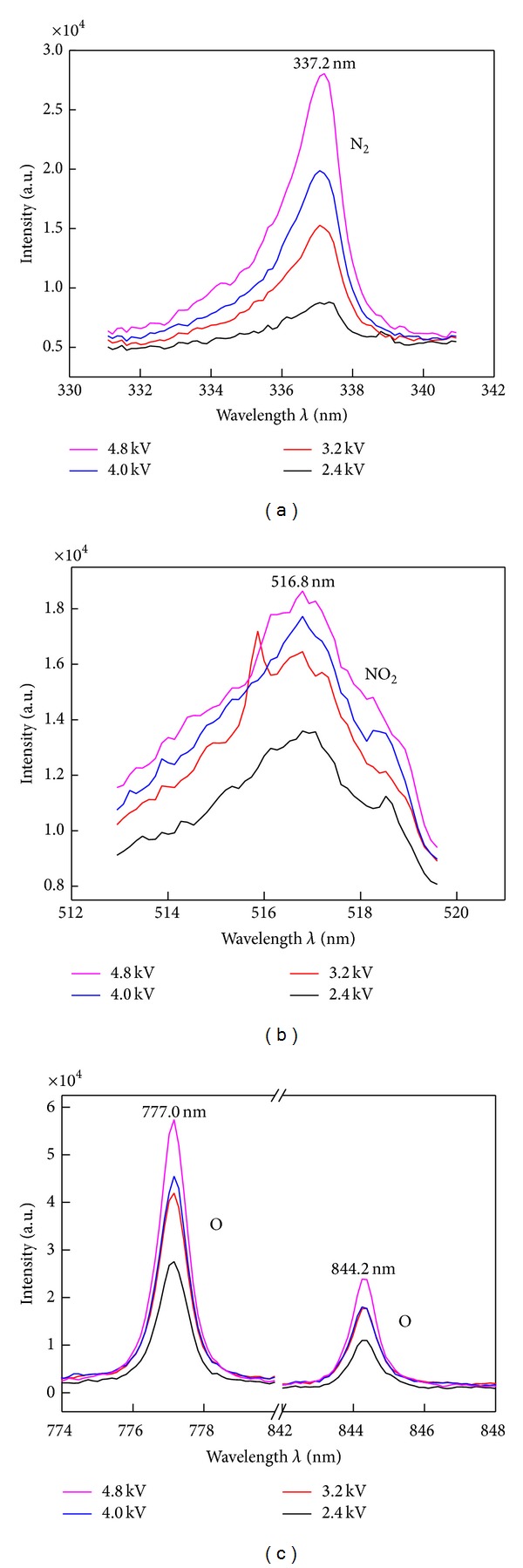
The characteristic spectra under different pulse voltage, identified at (a) 337.2 nm of N_2_ molecule, (b) 516.8 nm of NO_2_ molecule, and (c) 777.0 nm and 844.2 nm of O atom.

In order to clarify the influence of discharge energy, the characteristic spectra are selected at 337.2, 516.8, 777.0, and 844.2 nm for N_2_, NO_2_, and O, respectively.  Figure 5 shows the emission intensity variance under different inputting voltages. With input energy increasing, the emission intensity of the new species N_2_, NO_2_, and O all have increased.

According to *Einstein* spontaneous emission law in (1) [43], the species concentration *n*_*k*,*Z*_ is proportional to the emission intensity *I*_*ki*,*Z*_ as follows:

(1) $$\begin{array}{c} I_{ki,Z}=\frac{1}{4\pi }\frac{hc}{\lambda_{ki,Z}}n_{k,Z}A_{ki,Z}l. \end{array}$$

In (1), *A*_*ki*_ is the transition probability from energy level *k* to level *i* and *λ*_*ki*_ is the corresponding emission wavelength. *l* is the total plasma thickness. *h* is the *Planck*'s constant and *c* is the light velocity in vacuum. *n*_*k*,*Z*_ is the population in energy level *k* of the species.

Based on (1) and Figure 5, a qualitative conclusion can be made that the higher input energy has heightened NO removal efficiency to generate more new species of N_2_, NO_2_, O, and so forth.

In order to evaluate the plasma variance at different inputting energy, the plasma electron temperature *T*_e_ is analyzed according to the *Boltzmann* distribution law [44] under Local Thermodynamic Equilibrium (LTE) approximation as follows:

(2) $$\begin{array}{c} \frac{n_{k,Z}}{n_{Z}}=\frac{g_{k,Z}}{P_{Z}}\exp\left[-\frac{E_{k,Z}}{k_{B}T_{\text{e}}}\right]. \end{array}$$

In (2), *k*_*B*_ is the* Boltzmann's* constant. *P*_*Z*_ is related to the neutral or singly ionized atom state. *n*_*Z*_ is the total population density. *g*_*k*,*Z*_ and *E*_*k*,*Z*_ are the degeneracy and energy of the level *k*.

Since the emission intensity is detected with arbitrary unit, the electron temperature *T*_e_ is impossible to be directly calculated. In order to obtain the *T*_e_, it is deduced from the ratio of two emission lines of the same atoms with same ionization stage *Z*, which is applied based on (1) and (2) as

(3) I1I2=A1g1/λ1A2g2/λ2exp⁡(−E1−E2kBTe)or Te=−E1−E2kB(ln⁡(I1A2g2λ1)−ln⁡(I2A1g1λ2)).

Such procedure has eliminated the disturbance of arbitrary unit. The characteristic emission has been selected as the two O atom lines at 777.0 and 844.2 nm, due to their high signal-to-noise ratio. The emission constants are shown in Table 2.

**Figure 6:**
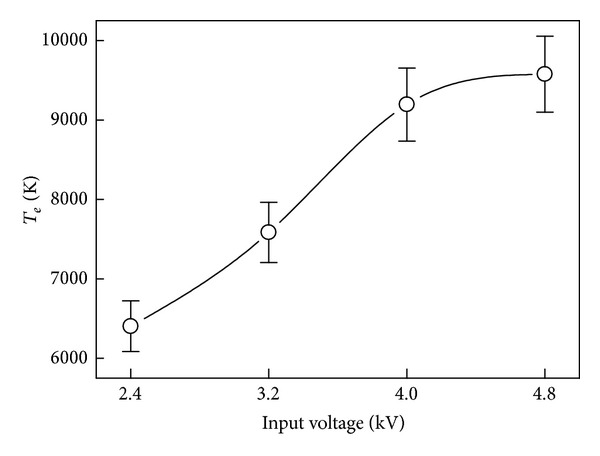
Plasma electron temperature *T*_e_ variance versus inputted voltage.

Plot of *T*_e_ versus the inputted voltage yields Figure 6. The obtained plasma electron temperature *T*_e_ is increasing with the voltage heightened. The 4.8 kV discharge has achieved higher *T*_e_ of about 9500 K, compared with that of 6403 K at 2.4 kV. Such *T*_e_ implies that the chemical reaction activation of the species in plasma is heightening for the NO removal.

It also should be noticed that NO transforming and removing process is difficult to be thoroughly detected by experimental methods, due to the complicated reactions in the plasma as shown in Figure 3. There has been a necessity to establish simulation model for analyzing the reaction dynamic.

### 3.2. Dynamic Simulation of NO Removal Process

To investigate the NO removal dynamic during and after discharge, the concentration variance of the *i*th species is modeled as a time varying differential equation [45], which includes the generating and losing reaction paths of this given *i*th species as in

(4) $$\begin{array}{c} \frac{dn_{i}}{dt}=-\sum_{i,j}k_{ij}n_{i}n_{j}+\sum_{p,q}k_{pq}n_{p}n_{q}, \end{array}$$

where, *n*_*i*_, *n*_*j*_, *n*_*p*_ and *n*_*q*_ are concentrations of the respective species and *k*_*ij*_ and *k*_*pq*_ are reaction rate coefficients of the losing and generating routines. Concentration loss of the *i*th specie has occurred in the reaction between species *i* and *j*, while specie *i* generation is determined by the reaction between species *p* and *q*.

With spatial homogeneity hypothesis of the discharge, the plasma is simulated in zero-dimensional scale. Space diffusion of electrons, NO, and other by-products has been neglected, and the concentration of electrons and gas molecules in the whole volume of the plasma are assumed to be uniform. The reaction paths and corresponding kinetic rate coefficients *k* are outlined in Table 3. For the electron-excited dissociation process, the rate constant *k* can be calculated by

(5) $$\begin{array}{c} k={\left(\frac{2}{m_{\text{e}}}\right)}^{1/2}\overset{\infty }{\underset{0}{\int }}E^{1/2}\sigma \left(E_{\text{e}}\right)f\left(E_{\text{e}}\right)dE_{\text{e}}. \end{array}$$

In which, *E*_e_ and *m*_e_ are the electron energy and mass. *f*(*E*_e_) is the electron distribution function, and *σ*(*E*_e_) is the corresponding collision cross section [46].

The species' reactions are modeled as discrete time-varying stiff differential equation system and have been solved by *Runge-Kutta* algorithm [47].

**Figure 7:**
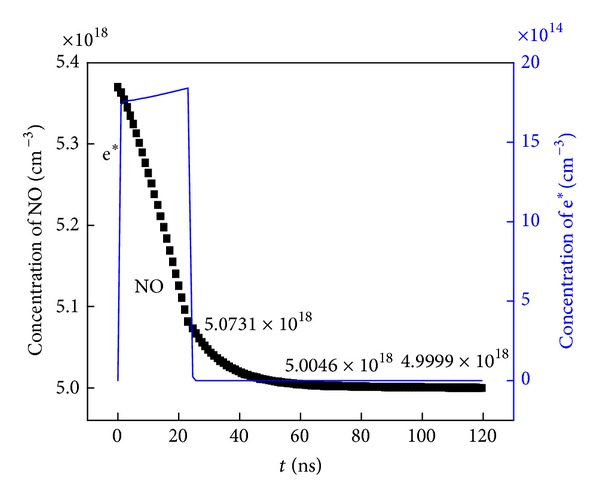
Concentration variance of NO molecules and electrons versus time. The concentration of NO is 5.37 × 10^18^/cm^3^ at 0 ns, 5.0731 × 10^18^/cm^3^ at 24.5 ns, 5.0046 × 10^18^/cm^3^ at 60 ns, and 4.9999 × 10^18^/cm^3^ at 120 ns.

NO concentration variance is shown in Figure 7, in which the concentration variance of electrons is also present. During pulse discharge, the concentration of NO is monotonically decreased. There was obvious NO removal effect, and about 5.5289% NO has been transformed into other species during discharge that lasted for 24.5 ns. After discharge, the electron concentration was assumed to be zero, whereas the NO concentration is also decreasing, and achieving 6.8045% NO removal efficiency after 60 ns as well as 6.8920% after 120 ns.

From the viewpoint of main reaction paths for NO removal, the collision dissociation reaction by electrons on NO molecules during discharge is occupied the major position, and determined the most part of the NO removal percentage. On the contrary, the by-product species and radicals are playing important role after discharge and contribute to a small part of the NO removal percentage.

**Figure 8:**
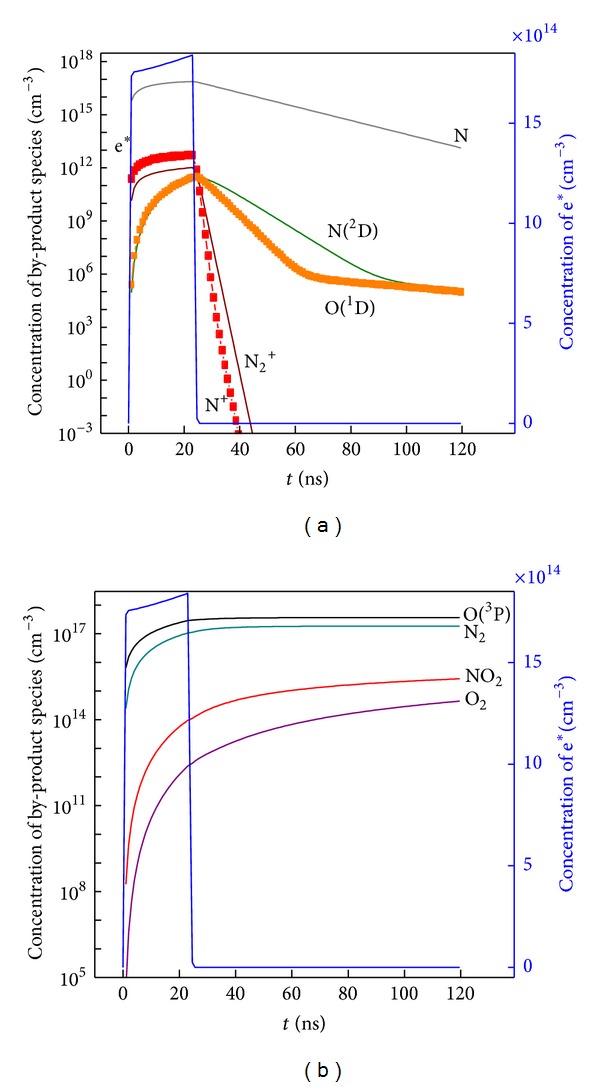
Concentration variance of by-product species and electrons versus time. (a) N^+^, N_2_^+^, O(^1^D), N(^2^D), N, and electron; (b) O(^3^P), N_2_, O_2_, NO_2_, and electron.

In order to clarify the effect of by-products on NO removal efficiency after discharge, the generating and losing dynamic process of O(^3^P), N_2_, NO_2_, O_2_, N, N(^2^D), N_2_^+^, N^+^, and O(^1^D) are shown in Figure 8. During discharge, concentrations of all the by-product species are increasing. When discharge is completed, some species such as N, N(^2^D), N_2_^+^, N^+^, and O(^1^D) are consumed as shown in Figure 8(a), while other species including O(^3^P), N_2_, and O_2_ as well as NO_2_ show an increasing trend of concentration in Figure 8(b). According to the law of conservation of matter, the generation of O(^3^P), N_2_, O_2_, and NO_2_ should be derived from the consumption of N, N(^2^D), N_2_^+^, N^+^, and O(^1^D). Also, it should take the different concentration magnitude order of all the species into consideration.

(*1) Role of O(* ^*3*^ *P) for NO Removal.* During discharge, large amount of O(^3^P) atoms have been accumulated. At 24.5 ns, the atom O(^3^P) concentration of 2.9648 × 10^17^/cm^3^ is higher than O(^1^D) of 3.0111 × 10^11^/cm^3^ with 10^6^ magnitude order. O(^3^P) has obviously played the major role in oxidizing NO to higher oxides. As consequence, the concentration increase of NO_2_ from 1.1101 × 10^14^/cm^3^ at 24.5 ns to final 2.6668 × 10^15^/cm^3^ has been mainly ascribed to the oxidation reaction between O(^3^P) and NO. Such oxidation reaction is ruled by

(6) O(P3)+NO+N2⟶NO2+N2;k=1.0×10−31 cm6 s−1.

But when it comes to the low magnitude order 10^15^/cm^3^ of NO_2_ produced after discharge, it can be deduced that there is a relatively small contribution of the oxidation reaction for the total 10^16^/cm^3^ NO removal after discharge from 5.0731 × 10^18^/cm^3^ at 24.5 ns to final 4.9999 × 10^18^/cm^3^ as shown in Figure 7.

There also has been O_2_ of 4.4522 × 10^14^/cm^3^ generated at 120 ns, which is obviously generated by bimolecular reactions between O(^3^P) atoms. But its concentration is relatively low and can be considered as neglectable reaction routine for NO removal.

(*2) Role of N Atoms for NO Removal.* Main part consumption of NO after discharge should be ascribed to generation of N_2_. For NO, the concentration decrease is in 10^16^/cm^3^ magnitude order. For N_2_, the concentration increase is also in the same 10^16^/cm^3^ orders from 1.1301 × 10^17^/cm^3^ at 24.5 ns to 1.8356 × 10^17^/cm^3^. In Figure 8(a), it can also be observed that the N atom of 7.0503 × 10^16^/cm^3^ at 24.5 ns which decreased to final 1.3421 × 10^13^/cm^3^ has shared the key reaction routine with NO to generate N_2_ after discharge. The N atoms have chemically substituted NO to produce N_2_ and O(^3^P) through the following reaction:

(7) N+NO⟶N2+O(P3); k=3.0×10−11 cm3 s−1.

From the viewpoint of concentration magnitude, other species such as N(^2^D), N_2_^+^, and N^+^ have little effect on NO removal after discharge.

**Figure 9:**
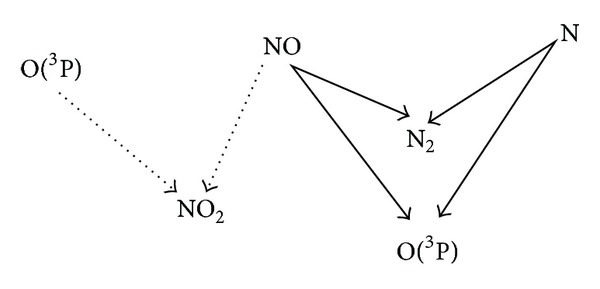
Diagram of main reaction paths for NO removal after discharge, including NO substituted by N as major reaction path (solid line) and NO oxidized by O(^3^P) as minor reaction path (dotted line).

Based on the above NO removal simulation after discharge, the N atoms in Reaction (7) are very effective in substituting NO, while the Reaction (6) of O(^3^P) atoms make a relatively small contribution by oxidizing NO. The main reaction paths and chemical species for NO removal after discharge are concluded in Figure 9.

### 3.3. Approaches for Improving the NO Removal Efficiency

(*1) Effect of Discharge Pulse Duration Width on NO Removal Efficiency.* Since the species of N and O(^3^P) atoms are both produced by electron impact dissociation of NO, the electron concentration has decided the dissociation rate of NO during discharge and has also affected the subsequent reactions between NO and the by-products during and after discharge.

**Figure 10:**
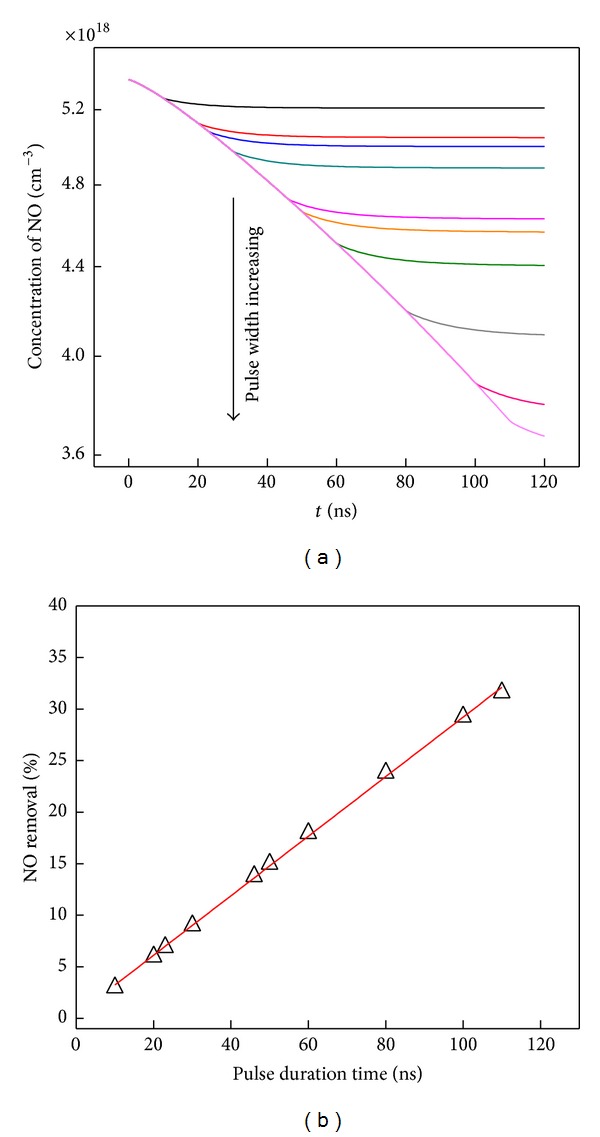
(a) NO concentration and (b) NO removal percentage achieved by adjusting pulse discharge duration width.

Adjustment of pulse discharge width can influence the electron accumulated concentration. In Figure 10(a), the pulse duration width is varied from 10 to 110 ns. The final NO concentrations are monotonically decreased with pulse width increasing.

Quantitatively, NO removal extent is calculated as

(8) $$\begin{array}{c} \eta =\frac{n{\left[\text{NO}\right]}_{t=0\;\text{ns}}-n{\left[\text{NO}\right]}_{t=120\;\text{ns}}}{n{\left[\text{NO}\right]}_{t=0\;\text{ns}}}\times 100\%. \end{array}$$

Linear increasing trend of NO removal extent has been presented in Figure 10(b); even 32% removal percentage is achieved at pulse duration of 110 ns. It is obvious that the electron concentration during discharge is accumulated with such a principle that its concentration is proportional to pulse duration width; the linear trend means that the NO removal efficiency is proportionally decided by the electron concentration in this simulation model. It also implies that the mechanism of NO removal during and after discharge is unchanged, though the inputted electron concentrations are different when pulse duration width varied.

(*2) Effect of N*_*2*_ *Additive on NO Removal Efficiency.* Based on the above analysis, the cost of NO removal is the discharge energy consumption with direct proportion principle. Then a question is brought out to find methods for more efficiently removing NO with less consumed discharge energy. Since the by-product species of N and O have played important roles on NO removal as shown in Figure 9, one practicable way is proposed to adjust gas ingredient in order to modify the reaction directions during and after discharge.

**Figure 11:**
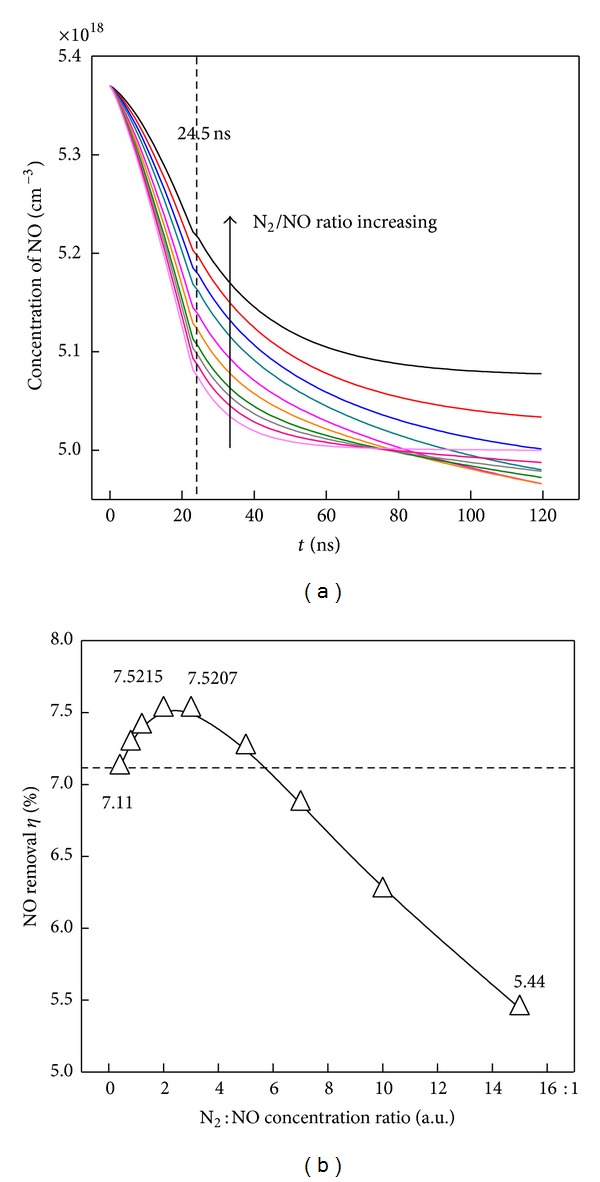
(a) The time evolution of NO concentration under different NO/N_2_ ratio; (b) NO removal percentage under different NO/N_2_ ratio.

In Figure 11(a), the N_2_ gas is mixed with NO. With N_2_ initial concentration increased, the NO concentration at 24.5 ns is heightened. It implies that the electron-collision dissociation reaction of NO has been weakened by increasing the N_2_ concentration. But the final NO removal percentage at 120 ns shows a trend as increasing first and then decreasing in Figure 11(b). The removal efficiency has been improved with mixed relatively low concentration of N_2_ and deteriorated by high concentration of N_2_ mixed.

In Figure 11(b), the highest removal extent is 7.52% at N_2_/NO ratio of 2 : 1, compared with 7.11% of no N_2_ mixed. When more N_2_ inputted, the removal extent is monotone decreased and even occurred as 5.44% at N_2_/NO ratio of 15 : 1. Such result shows that the by-products have affected or even improved the reaction dynamic after discharge, though electron collision dissociation of NO during discharge have been weakened when N_2_ mixed.

**Figure 12:**
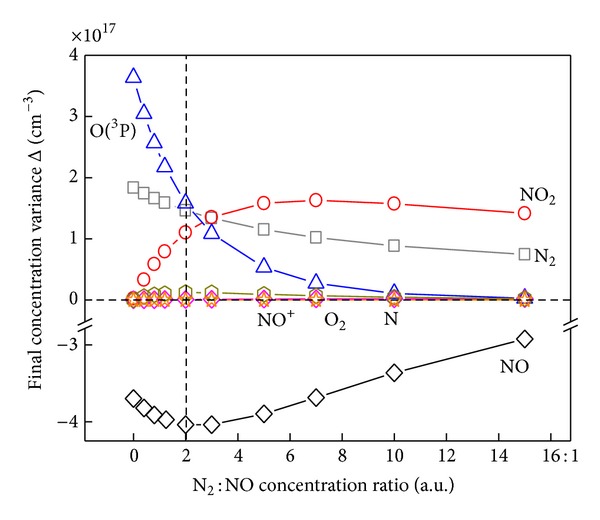
The final concentration variance Δ of the main species under different N_2_/NO ratio.

In order to clarify the improving mechanism by mixed N_2_, the final concentration variance Δ of the main species under different N_2_/NO ratio is shown in Figure 12. By defining *n* ^*i*^ as the concentration of *i*th species, the Δ_*i*_ is defined as

(9) $$\begin{array}{c} \Delta_{i}=n_{t=0\;\text{ns}}^{i}-n_{t=120\;\text{ns}}^{i}. \end{array}$$

In Figure 12, the removal variance of NO is accompanied with the generation variance of N_2_ and NO_2_ as well as O(^3^P) and varied in same magnitude order. With N_2_/NO ratio increased, the final generation concentrations of N_2_ and O(^3^P) are monotonically decreased, while that of NO_2_ is first increasing and then decreasing. Variance of other species such as NO^+^, O_2_, and N can be neglected.

It is obvious that the higher the N_2_/NO concentration ratio is, the less the electrons are consumed by NO due to the decreased collision probability. Consequently, the concentration of N and O(^3^P) atoms dissociated from NO as ruled in Reaction (10) would be decreased. Consider

(10) e∗+NO⟶N+O(P3)+e; k=8.5×10−10 cm3 s−1.

But there is also a compensative path for N atom loss according to Reaction (11) by electron collision on the N_2_ molecules:

(11) $$\begin{array}{c} {\text{e}}^{*}+{\text{N}}_{2}⟶\text{N}+\text{N}+\text{e};\;k=2.0\times 10^{-11}\;{\text{cm}}^{3}{\text{s}}^{-1}. \end{array}$$

The two reactions are competitive with each other for consuming electrons, during which the dissociation energy of NO is found to be smaller than that of N_2_. For the former, the dissociation energy of N–O bond is 6.496 eV. For the latter, the N–N bond is dissociated at 9.76 eV [48]. As a result, the reaction rate coefficients are different, and the N_2_ dissociation rate coefficient of 2.0 × 10^−11^ cm^3^ s^−1^ is lower at the magnitude order of 10^−1^ than 8.5 × 10^−10^ cm^3^ s^−1^ of NO. The N and O(^3^P) atom concentrations are predicted to present a decreasing trend though more N_2_ mixed at higher N_2_/NO ratio.

**Figure 13:**
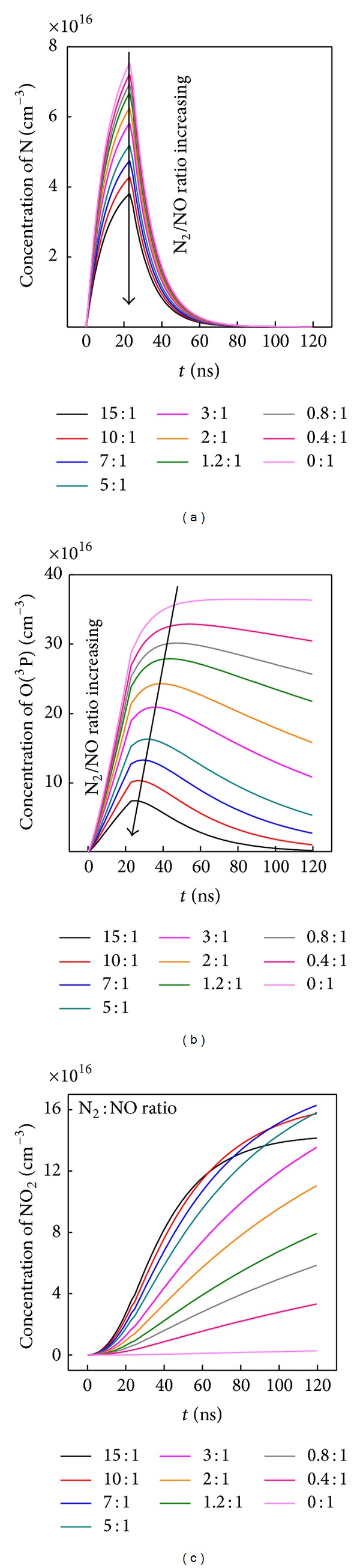
Concentration evolution versus time of (a) N atoms, (b) O(^3^P) atoms, and (c) NO_2_ molecules under different N_2_/NO mixing ratio. The arrow direction represents the N_2_/NO mixing ratio increase.

This phenomenon is verified based on the time-evolution of main species' concentrations in Figure 13. With more N_2_ mixed, the N concentration at 24.5 ns is monotonically decreased in Figure 13(a). For the O(^3^P) atoms, its concentration is also decreased in Figure 13(b).

Also in Figure 13(b), O(^3^P) accumulating process has presented longer time than the discharge width of 24.5 ns. With N_2_/NO ratio increased, the accumulating process has been shortened. The reason is that the O(^3^P) is not only derived from the electron collision dissociation in Reaction (10) during discharge, but can also be generated from Reaction (12) as

(12) N+NO⟶N2+O(P3); k=3.0×10−11 cm3 s−1.

Since the N atom concentration generated during discharge has been weakened by increasing N_2_/NO ratio, the Reaction (12) for further generating O(^3^P) between N and NO has sequently been weakened. Reaction (10) during discharge has played more and more important roles for O(^3^P) generation; therefore, the accumulating process presents a shorter time at higher N_2_/NO ratio in Figure 13(b). As another result, the generated N_2_ concentration, which operated according to the Reaction (12), has also been attenuated. Consequently, concentrations of the generated N_2_ and O(^3^P) both have shown monotonically decreasing trends in Figure 12.

When it comes to NO_2_, the NO_2_ yielded from Reaction (13) is determined by O(^3^P) and N_2_:

(13) O(P3)+NO+N2⟶NO2+N2;k=1.0×10−31 cm3 s−1.

Raising reactant concentrations usually makes the reaction operates at a higher rate by increasing collision probability per unit time. More N_2_ additive has accelerated Reaction (13) for oxidizing NO into more NO_2_. With N_2_/NO mixing ratio increased, the formation of NO_2_ is achieved to be maximal at N_2_/NO of 8 : 1. When too more N_2_ molecules are inputted, the O(^3^P) atom concentration is remarkably decreased according to the above analysis; then the Reaction (13) becomes slow. As shown in Figure 13(c), NO_2_ concentration is decreased when too more N_2_ are inputted at the N_2_/NO ratio of 10 : 1 and 15 : 1.

Overall, the NO removal is decided by the electron collision dissociation during discharge and the substitution reaction or oxidation process during and after discharge. The optimal N_2_/NO mixing ratio is 2 : 1. In such a ratio, the total generated O/N_2_/NO_2_ concentration is maximum, which can not only compensate the disadvantage of competitive electron consumption by the added N_2_ during discharge as shown in Figure 11(a) but also heighten the total removal percentage in Figure 11(b). By mixing N_2_ gas at appropriate concentration ratio, the NO removal process has been more efficiently achieved, without depositing additional discharge energy.

The optimal condition of pulsed-discharged plasma technique for NO pollutant gas removal has been quantitatively obtained in this paper through the numerical simulation method. Such results can be applied for NO gas remediation in power plants, automobile exhaust treatment, and so on.

## 4. Conclusion

This paper discusses the NO removal mechanism in pulse-discharged plasma. Emission spectra diagnosis indicates that the NO has transformed into O, N, N_2_, NO_2_, and other by-products, and the higher the discharge voltage is, the more the NO removed is. The plasma electron temperature *T*_e_ is deduced as 9500 K at voltage of 4.8 kV inputted. The chemical reaction activation of the species has been heightened in such high *T*_e_. In order to clarify the complicated NO removal dynamic process, zero-dimensional numerical simulation indicates that the main reaction paths during pulsed discharge are the electron collision dissociation of NO into N and O atoms, and followed single substitution of N on NO to form N_2_. After discharge, the single substitution between N and NO to form N_2_ also plays the major role, compared with the NO_2_ formation by oxidizing NO. This model also implies that the NO removal mechanism is almost the same when pulse discharge with different duration width is operated.

Furthermore, the dynamic model gives particular notice on the N_2_ additive for improving NO removal efficiency. With the N_2_/NO ratio increased, the maximum concentration of NO_2_ is achieved at ratio of 8 : 1, but the N_2_ and O(^3^P) concentrations are decreasing remarkably due to the modified reaction directions. There has an optimal N_2_/NO concentration ratio as 2 : 1 to achieve the maximal NO removal efficiency. At such a ratio, the total generated O/N_2_/NO_2_ concentration is maximum, which cannot only compensate the competitive electron consumption by N_2_ additive during discharge but also heighten the total removal percentage than that without N_2_ mixed.

Such preliminary results indicate that the nonthermal pulse-discharged plasma technique for NO pollutant gas control must necessarily include adequate additive gas, such as the N_2_, for low-costly improving the removal efficiency.

## Figures and Tables

**Table 1:** Emission spectra and transition channel diagnosed from the NO emission lines.

*λ* /nm	Transition channel	Species
236.1	A^2^∑ → X^2^∏ (0-1)	NO
247.3	A^2^∑ → X^2^∏ (0–2)	NO
258.9	A^2^∑ → X^2^∏ (0–3)	NO
271.4	A^2^∑ → X^2^∏ (0–4)	NO
280.0	A^2^∑ → X^2^∏ (1–6)	NO
295.2	A^2^∑ → X^2^∏ (1–7)	NO
315.8	C^3^∏_*u*_ → B^3^∏_*g*_ (1-0)	N_2_
337.2	C^3^∏_*u*_ → B^3^∏_*g*_ (0-0)	N_2_
357.3	C^3^∏_*u*_ → B^3^∏_*g*_ (0-1)	N_2_
375.1	C^3^∏_*u*_ → B^3^∏_*g*_ (1–3)	N_2_
380.0	C^3^∏_*u*_ → B^3^∏_*g*_ (0–2)	N_2_
391.2	B^2^∑_*u*_^+^ → X^2^∑_*g*_^+^ (0-0)	N_2_^+^
399.5	C^3^∏_*u*_ → B^3^∏_*g*_ (1–4)	N_2_
405.6	C^3^∏_*u*_ → B^3^∏_*g*_ (0–3)	N_2_
426.5	B^2^∑_*u*_^+^ → X^2^∑_*g*_^+^ (0-1)	N_2_^+^
434.0	C^3^∏_*u*_ → B^3^∏_*g*_ (0–4)	N_2_
441.4	C^3^∏_*u*_ → B^3^∏_*g*_ (3–8)	N_2_
464.5	B^2^∑_*u*_^+^ → X^2^∑_*g*_^+^ (1–3)	N_2_^+^
470.7	B^2^∑_*u*_^+^ → X^2^∑_*g*_^+^ (0–2)	N_2_^+^
493.4	A^2^B_1_ → X^2^A_1_(1,5, 0-0,0, 0)	NO_2_
516.8	A^2^B_1_ → X^2^A_1_(1,4, 0-0,0, 0)	NO_2_
540.4	A^2^B_1_ → X^2^A_1_(1,3, 0-0,0, 0)	NO_2_
552.3	A^2^B_1_ → X^2^A_1_(3,0, 0-0,0, 0)	NO_2_
568.0	A^2^B_1_ → X^2^A_1_(1,2, 0-0,0, 0)	NO_2_
5884	A^2^B_1_ → X^2^A_1_(2,0, 0-0,0, 0)	NO_2_
597.3	A^2^B_1_ → X^2^A_1_(1,1, 0-0,0, 0)	NO_2_
602.2	A^2^B_1_ → X^2^A_1_(0,0, 1-0,0, 0)	NO_2_
656.4	2*s*2*p* ^2^(^4^P)3*s* → 2*s* ^2^2*p*4*s*	N^+^
674.6	2*s* ^2^2*p* ^2^(^3^P)4*d* → 2*s* ^2^2*p* ^2^(^3^P)3*p*	N
715.5	2*s*2*p* ^2^(^4^P)3*s* → 2*s* ^2^2*p*4*s*	N^+^
746.8	2*s* ^2^2*p* ^2^(^3^P)3*p* → 2*s* ^2^2*p* ^2^(^3^P)3*s*	N
777.0	2*s* ^2^2*p* ^3^(^4^S°)3*p* → 2*s* ^2^2*p* ^3^(^4^S°)3*s*	O
821.5	2*s* ^2^2*p* ^2^(^3^P)3*p* → *2s* ^2^2*p* ^2^(^3^P)3*s*	N
844.2	2*s* ^2^2*p* ^3^(^4^S°)3*p* → 2*s* ^2^2*p* ^3^(^4^S°)3*s*	O
867.6	2*s* ^2^2*p*5*s* → 2*s* ^2^2*p*4*p*	N^+^

**Table 2:** Spectra constants of O atoms at 777.0 and 844.2 nm [20].

*E*_*k*_/eV	*λ*_*k*_/nm	*A*_*k*_ × *g*_*k*_/s^−1^
10.740931	777.0	2.58 × 10^8^
10.988861	844.2	1.61 × 10^8^

**Table 3:** Reaction paths and the corresponding reaction rate coefficients [21–26].

Reaction path	*k*/cm^3^s^−1^
e* + N_2_ → N_2_^+^ + 2e	2.4 × 10^−12^
e* + N_2_ → N + N + e	2.0 × 10^−11^
e*+ NO → N + O(^3^P) + e	8.5 × 10^−10^
e*+ NO → NO^+^ + 2e	1.1 × 10^−10^
e* + NO^+^ → N + O(^3^P)	8.2 × 10^−9^
e* + N_2_^+^→ N_2_	4.0 × 10^−12^
e* + NO^+^→ O(^3^P) + N(^2^D)	4.3 × 10^−7^
N^+^ + NO → N_2_^+^ + O(^3^P)	5.0 × 10^−11^
N^+^ + O_2_ → NO^+^ + O(^3^P)	2.6 × 10^−10^
N_2_^+^ + N → N^+^ + N_2_	1.0 × 10^−11^
N_2_^+^ + O(^3^P) → NO^+^ + N	1.4 × 10^−10^
N + NO_2_ → N_2_ + O(^3^P) + O(^3^P)	9.1 × 10^−13^*
O(^3^P) + NO + N_2_ → NO_2_ + N_2_	1.0 × 10^−31^
N_2_(A) + O_2_ → N_2_ + O(^3^P) + O(^3^P)	2.54 × 10^−12^
N_2_(A) + NO → NO + N_2_	7.0 × 10^−11^
N_2_(A) + N_2_ → N_2_ + N_2_	3.0 × 10^−18^
N_2_(A) + NO_2_→ NO + O(^3^P) + N_2_	1.0 × 10^−12^
NO_2_ + O(^3^P) → NO + O_2_	9.1 × 10^−12^
O(^3^P) + O(^3^P) + O_2_ → O_2_ + O_2_	6.7 × 10^−33^*
N_2_(B) + O_2_→ N_2_(A) + O_2_	3.0 × 10^−10^
N(^2^D) + O_2_→ NO + O(^1^D)	6.0 × 10^−12^
N(^2^D) + NO → N_2_ + O(^3^P)	4.5 × 10^−11^
O(^1^D) + NO → N + O_2_	8.5 × 10^−11^
e* + N_2_ → N^+^ + N + 2e	2.4 × 10^−17^
e* + N_2_ → N_2_(A) + e	1.1 × 10^−10^
e* + N → N^+^ + 2e	1.1 × 10^−10^
e* + N^+^ → N	3.5 × 10^−12^
e* + NO^+^ → NO	4.0 × 10^−12^
N^+^ + NO → NO^+^ + N	5.1 × 10^−10^
N^+^ + NO_2_ → NO^+^ + NO	5.0 × 10^−10^
N_2_^+^ + O_2_ → NO^+^ + NO	1.0 × 10^−17^
N_2_^+^ + NO → NO^+^ + N_2_	3.3 × 10^−10^
N + NO → N_2_ + O(^3^P)	3.0 × 10^−11^
N + NO_2_ → 2NO	6.0 × 10^−13^
N + O_2_ → NO + O(^3^P)	8.9 × 10^−17^
N_2_(A) + O(^3^P) → NO + N(^2^D)	7.0 × 10^−12^
N_2_(A) + N_2_(A) → N_2_ + N_2_(C)	1.6 × 10^−10^
N_2_(A) + O(^3^P) → N_2_ + O(^1^D)	2.1 × 10^−11^
O(^1^D) + O_2_ → O(^3^P) + O_2_	8.0 × 10^−12^
O(^3^P) + O(^3^P) + N_2_ → O_2_ + N_2_	3.0 × 10^−33^*
N_2_(B) + N_2_ → N_2_(A) + N_2_	3.0 × 10^−11^
N_2_(B) + NO → N_2_(A) + NO	2.4 × 10^−10^
N_2_(C) + N_2_ → N_2_(B) + N_2_	1.0 × 10^−11^
N(^2^D) + O_2_ → NO + O(^3^P)	1.5 × 10^−12^
N(^2^D) + N_2_ → N + N_2_	6.0 × 10^−15^
O(^1^D) + N_2_ → O(^3^P) + N_2_	2.6 × 10^−11^

*Its unit is cm^6^s^−1^.
